# MEETING THE CHALLENGE OF AGE-INCLUSIVE EDUCATION: FOCUS GROUP PERSPECTIVES

**DOI:** 10.1093/geroni/igad104.0643

**Published:** 2023-12-21

**Authors:** Susan Whitbourne

**Affiliations:** University of Massachusetts Boston, Boston, Massachusetts, United States

## Abstract

Through a series of 16 virtual focus groups involving a national sample of 78 faculty, staff, and students, a set of challenges and related strategies for meeting those challenges were identified based on the ICCS responses from campus constituent groups of faculty, staff, and students. Qualitative analysis of focus group transcripts in the areas of teaching/learning, personnel, and student affairs produced 969 strategy statements that were then collapsed into thematic groupings based on the challenges they were intended to address. Major challenges and their relevant areas included the following: Long-term employees often feel undervalued (personnel), non-traditional age and older students often feel that career services do not meet their needs, and non-traditional age and older students often feel excluded or disregarded in the post-secondary classroom. Focus group members suggested strategies in the area of personnel that included greater recognition of accomplishments of long-term employees. In student affairs, they suggested such strategies as providing more resources for advising and career services. Teaching and learning strategies included incentivizing faculty to develop age-inclusive practices and promoting age-friendly classroom and course offerings. These findings suggest ways for campuses to enhance age-inclusive practices through specific actions, but their feasibility and impact remain necessary to evaluate.

